# Recent advances in the understanding and management of underactive bladder

**DOI:** 10.12688/f1000research.13660.1

**Published:** 2018-04-10

**Authors:** Su-Min Lee, Hashim Hashim

**Affiliations:** 1 Bristol Urological Institute, Southmead Hospital, Southmead Road, Bristol, BS10 5NB, UK

**Keywords:** underactive bladder, detrusor underactivity, LUTS

## Abstract

Underactive bladder (UAB) is an important and complex urological condition resulting from the urodynamic finding of detrusor underactivity. It can manifest in a wide range of lower urinary tract symptoms, from voiding to storage complaints, and can overlap with other conditions, including overactive bladder and bladder outlet obstruction. However, UAB continues to be poorly understood and inadequately researched. In this article, we review the contemporary literature pertaining to recent advances in defining, understanding, and managing UAB.

## Introduction

Underactive bladder (UAB) is an important urological condition that can manifest with a range of lower urinary tract symptoms (LUTSs), including reduced urinary flow rate and the feeling of incomplete bladder emptying because of an increased post-void residual (PVR) volume. Some patients may experience storage symptoms, similar to those of patients with an overactive bladder, including frequency, urgency, nocturia, and incontinence, due to a high PVR volume.

Evidence of detrusor underactivity (DU) in patients undergoing urodynamic investigation for LUTSs may be present in as many as 48% of older (>70 years of age) men and 45% of older women ^1, 2^. A recent cross-sectional survey found that only 11% of the public had heard of UAB, despite its prevalence ^3^. Within medicine, UAB has received little research attention and continues to be poorly understood; however, it has been garnering attention in more recent times. Therefore, this review aims to highlight recent advances in defining, understanding, and managing UAB.

## Definition

No universally accepted definition currently exists for UAB. In addition, a variety of terms have been used in the literature to describe the condition, including DU, ‘non-obstructive voiding dysfunction’, ‘voiding dysfunction’, or ‘detrusor voiding insufficiency’, creating further confusion ^4^.

In 2002, the International Continence Society (ICS) defined DU as “a contraction of reduced strength and/or duration, resulting in prolonged bladder emptying and/or failure to achieve complete bladder emptying within a normal time span” ^5^. This definition, based on expert opinion, is formed on the basis of urodynamics characterised by low-pressure or poorly sustained contractions with associated poor flow. Unfortunately, it lacks quantification and fails to include values for normal time spans and reduced strength. Furthermore, it focuses only on detrusor contraction during urodynamics, which forms only part of a potentially multifactorial problem.

For this reason, authors have suggested that UAB should be defined on the basis of clinical symptoms, similar to the way in which overactive bladder is defined ^1, 6^. Chapple *et al*. proposed a definition for UAB as “a symptom complex suggestive of DU and is usually characterised by prolonged urination time with or without a sensation of incomplete bladder emptying, usually with hesitancy, reduced sensation on filling, and a slow stream” ^7^.

Most recently, a think tank commissioned by the International Consultation on Incontinence Research Society (ICI-RS) in 2014 defined UAB as “the perception of detrusor underactivity, characterised by symptoms of prolonged voiding, hesitancy, slow and/or intermittent stream, and/or sensation of incomplete emptying. It is not a pathophysiologic or functional statement” ^8^. The term ‘DU’ is reserved purely for urodynamic findings, as defined by the ICS.

Given that patients present to their doctor seeking treatment for these UAB symptoms, the overall definition needs to be simplified for primary care physicians, without the need for invasive urodynamics. Therefore, participants of the 2nd International Congress of Underactive Bladder (CURE-UAB 2) in 2015 chose to endorse the Chapple *et al*. ^7^ definition for UAB, supplemented with additional symptoms noted to be more common in patients with DU as opposed to bladder outlet obstruction (BOO) (that is, palpable bladder, always straining to void, enuresis or stress urinary incontinence, or a combination of these) ^9^. The ongoing need for a universal definition for UAB is clear, and future definitions must represent a symptom complex as opposed to a purely urodynamic modality.

## Clinical symptoms

Identifying clinical symptoms for UAB is challenging because of considerable overlap with those of overactive bladder and symptoms associated with BOO ^10^. Storage symptoms may include frequency, urgency, and nocturia but also the opposite: poor sensation and less frequent voiding leading to overflow incontinence ^11^.

Recently, Gammie *et al*. ^12^ described signs and symptoms of DU in a large cohort undergoing urodynamics. Compared to those with normal tests, men with DU had statistically higher occurrence of decreased/interrupted stream (56% versus 30%), hesitancy (51% versus 26%), feeling of incomplete bladder emptying (36% versus 22%), palpable bladder (14% versus 1.1%), and absent/decreased sensation (13% versus 3.0%). Women with DU had statistically higher rates of decreased/interrupted stream (29% versus 4.0%), hesitancy (28% versus 9.1%), feeling of incomplete emptying (28% versus 20%), palpable bladder (3.3% versus 1.5%), absent/decreased sensation (4.3% versus 0.8%), enuresis (12% versus 8.4%), and impaired mobility (13% versus 2.8%) ^12^.

Compared with those with BOO, men with DU reported statistically greater rates of sexual dysfunction (41% versus 26%), stress incontinence (25% versus 3.7%), enuresis (21% versus 1.8%), palpable bladder (14% versus 0.6%), absent/decreased sensation (14% versus 0%), always straining to void (8.0% versus 0.5%), poor bowel control (5.4% versus 0.6%), and feeling of incomplete bowel emptying (8.6% versus 0.6%) ^12^. Lower rates of poor stream (56% versus 82%), hesitancy (51% versus 69%), and urgency (30% versus 45%) were seen in men with DU versus men with BOO.

Uren *et al*. ^13^ used semi-structured interviews to understand the experience of patients with urodynamically diagnosed DU. A wide range of LUTSs were described, consistent with those described by Gammie *et al*. ^12^. In addition, nearly 40% of patients report recurrent urinary tract infections, over 50% described a history of self-catheterisation, and approximately 15% had previous episodes of acute urinary retention. DU led to a broad and profound disruption to quality of life.

Observations from these studies may help in the identification of UAB patients and differentiation from symptoms secondary to BOO. However, these studies were aided by a clear urodynamic diagnosis, and there remains significant overlap between the conditions; diagnosis based on symptoms alone would prove challenging even to experts. In addition, the underlying cause of UAB was not subclassified in these studies; clarification of symptoms at the time of urodynamic diagnosis may aid clinicians in determining UAB aetiology and focus management therapies appropriately. For example, patients with diabetic cystopathy may experience reduced sensation of bladder fullness, which may be reflected during urodynamic testing ^14^. Symptomatic diagnosis in UAB is complex, and the development of non-invasive surveys and tests to characterise UAB has been recognised by several authors; there is ongoing work within this research area ^1, 12^.

## Pathophysiology

Micturition is a complicated process and abnormalities along the pathway—including physical structures, such as muscle and mucosa, sensory (afferent) or efferent portions of the micturition reflex, or the central nervous system or a combination of these—can lead to impaired detrusor function and UAB. The aetiology of DU is unclear but is thought to arise from a number of possible pathologies and often is subclassified into three types on the basis of possible disease mechanisms: idiopathic, myogenic, and neurogenic groups.

### Idiopathic

UAB associated with aging and a decrease in bladder contractility has been defined as idiopathic. These patients have no anatomic or functional BOO, no neuropathy, low or absent detrusor pressure, a maximum flow rate of less than 10 ml/s and a large PVR volume (more than 150 ml) ^15^.

Chancellor first suggested that untreated OAB can progress to UAB ^16^. Structural changes in OAB may lead to alterations in bladder muscle, mucosa, or connective tissue, causing impaired contractility ^17^. Alternatively, Drake *et al*. ^18^ highlighted the role of unregulated autonomous bladder micromotions. Sympathetic outflow to the bladder lowers muscle tone, reducing phasic pressure changes and facilitating urine storage. Detrusor overactivity arises when areas of the bladder become denervated and express autonomous micromotility. In mild cases of denervation, voiding is unaffected because a normal bladder can trigger synchronous micromotions in denervated areas. However, as denervation worsens, voiding becomes increasingly inefficient, leading to underactivity ^18^.

### Neurogenic

Neurogenic causes of DU may arise anywhere along the micturition pathway, including bladder afferent and efferent pathways and the lumbosacral spinal cord (spinal micturition centre) ^19^. Intact sensory (afferent) input, which monitors bladder filling volume, is required to generate effective voluntary detrusor contraction; defective signals may result in inadequate contraction strength ^20^. Alternatively, a lack of contractile stimulus (that is, acetylcholine) or tissue responsiveness may cause UAB. Reduced acetylcholine release or increased degradation can lead to a smaller contractile stimulus and may arise from the pontine micturition centre or impaired transmitter release ^21^.

A number of neurological conditions may lead to neurogenic DU with selected conditions described here. Patients with acute cerebrovascular accidents (CVAs) may develop temporary or permanent bladder dysfunction, and 50% develop acute retention; three-quarters of these patients will have no detrusor contraction present ^22^. The location appears to be important, and medullary infarcts have higher DU rates than do pontine infarcts ^23^. In particular, lateral medullary infarcts have higher DU rates because of damage to descending pathways of the pontine micturition centre. In contrast, multiple sclerosis is caused by focal demyelination of the central nervous system and may occur at multiple levels; however, as with CVA, location is important, and patients with cervical cord or pontine lesions are more likely to develop DU ^24, 25^. Bladder function can also be affected by injuries to the spinal cord, cauda equina or peripheral sacral nerves, through trauma or infection. Injuries to the cauda equina or parasympathetic (S2–S4) nerve roots may lead to decreased bladder contraction and elevated urethral muscle resistance ^26^.

Smith *et al*. ^27^ suggest that, instead of defects in the afferent or efferent micturition limbs (or both), defective central volume sensation contributes to DU. A review of urodynamic traces found that, compared with other groups, patients with DU have higher overall bladder capacity and bladder wall stress when they reach a strong desire to pass urine on urodynamics. However, they voided smaller volumes and had elevated PVR volumes, suggesting that diminished central sensitivity to volume afferent activity (as opposed to impaired afferent generation) may contribute to the development of idiopathic UAB.

Finally, UAB may arise through iatrogenic causes, particularly in patients undergoing pelvic surgeries (for example, abdominoperineal resection, radical hysterectomy, and radical prostatectomy). Terminal branches of the pelvic plexus innervate the bladder, and it has been suggested that shear injury at the time of operation might lead to neurogenic UAB ^28^. In particular, denervation due to iatrogenic injury to the nerves supplying the bladder trigone (for example, during radical prostatectomy) may occur ^29^. Alternatively, Kitta *et al*. proposed a multifactorial cause of iatrogenic UAB, including temporary blood flow reduction due to surgery, injury to bladder innervation, pain, and anxiety leading to reduced pelvic floor relaxation ^30^.

### Myogenic

Myogenic DU can refer to any abnormality within the detrusor muscle structure or the intrinsic properties of the myocytes. This may include alterations in the excitation-contraction mechanisms within the muscle, changes in ion storage and transport, or changes in calcium storage and energy generation ^21^. As these muscle properties change, contractile power may be attenuated, leading to DU. Osman *et al*. include BOO and diabetes mellitus as potential myogenic causes ^1^.

Despite this subclassification based on possible disease aetiology, the pathophysiology of DU may be multifactorial. In fact, in a study of women, Brown *et al*. ^31^ compared urodynamic findings based on presumed DU aetiology, including neurologic, idiopathic, and cardiovascular cohorts. The authors did not demonstrate distinct clinical and urodynamic parameters between the groups, suggesting a multifactorial DU aetiology.

### Diabetes mellitus

Bladder dysfunction may occur in patients with diabetes mellitus, and the term diabetic cystopathy is used to describe symptoms of decreased bladder sensation and contraction along with increased bladder capacity and PVRs ^32^. The cause of diabetic cystopathy is likely multifactorial, through peripheral neuropathic, microvascular, or myogenic changes or a combination of these ^31^.

The mechanism behind diabetic complications was proposed by Brownlee ^33^. Hyperglycaemia leads to increased oxidation of glucose during the Krebs cycle, and electrons are donated to molecular oxygen to generate reactive oxygen species (ROS) superoxide ^34^. This leads to the activation of specific damaging pathways (that is, increased production of advanced glycation end products, protein kinase C signalling, and movement through the hexosamine and polyol pathways), ending with deranged gene expression changes and further oxidative stress ^33^. These stresses can lead to a polyneuropathy of the sensory and autonomic nerve fibres of the bladder.

In addition, polyuria may lead to myogenic changes. In a mouse bladder model, sucrose-induced diuresis leads to bladder hypertrophy and increased contractility, capacity, and compliance ^35^. However, the bladder transitions to decreased micturition and increased residual volumes at a later stage, suggesting that polyuria can alter the structure and function of the bladder ^36^.

## New management strategies for underactive bladder

The primary aims of the management of patients with UAB should include prevention of upper urinary tract damage, avoidance of overdistension, and reduction of PVR urine through improvements in bladder function and emptying. These aims overlap with the management of chronic urinary retention caused by BOO. Given that many urological patients will never undergo invasive urodynamic testing, both UAB and BOO may contribute to a raised PVR. The American Urological Association has published guidance and a treatment algorithm for such patients with chronic retention, and patients are subclassified on the basis of high-risk features (that is, hydronephrosis, chronic kidney disease stage 3, or recurrent infections and symptomatology) ^37^. On the basis of these features, patients may undergo observation, treatment, or further testing through invasive urodynamics.

We focus here on patients with formal UAB diagnosis. In these patients, existing management strategies are often limited and inadequate. In asymptomatic patients with existing UAB but no high-risk features, a period of observation may be suitable prior to any additional treatment ^37^. In those presenting with symptoms, behavioural modifications (such as timed voiding and double voiding) or intermittent or indwelling catheterisation is typically utilised. Pharmacological management is similarly poor, and there is no clear beneficial evidence for parasympathomimetic agents, intravesical prostataglandin E _2_ (PGE _2_), or alpha-adrenergic blockers ^38– 42^.

Surgical treatment options, including transurethral resection of the prostate (TURP) ^43, 44^, holmium laser enucleation of the prostate (HoLEP) ^45^, and intrasphincteric botulinum toxin ^46, 47^, have demonstrable short-term benefits but limited long-term benefit. In a small cohort of nine patients with DU and eight with acontractility, Lomas and Krambeck demonstrated that eight (89%) with DU and five (63%) with acontractility were left catheter-free at a median 50 and 39 months, respectively ^45^. Reducing bladder outflow obstruction can aid these patients, presumably allowing them to void through straining. However, these studies are retrospective in nature and do require further prospective randomised evaluation.

Sacral neuromodulation (SNM) has been shown to be effective in selected patients; in successful patients, SNM can increase voided volume and reduce PVRs ^48^, but overall response rate is varied, ranging from 33% to 90% ^49^. The single randomised placebo-controlled trial of SNM for urinary retention showed that 69% of patients with implants eliminated catheterisation at 6 months and that an additional 14% had more than 50% reduction in volume per catheterisation; successful results were seen in 83% versus 9% in the control group ^50^.

A number of novel therapies that could benefit the patient with UAB are being evaluated. Gene therapy has potential in restoring bladder function in neurogenic-type DU. Rats with induced diabetes and voiding dysfunction have reduced nerve growth factor (NGF) in the L6 to S1 dorsal root ganglia ^51^. Gene therapy using replication-defective herpes simplex virus vectors carrying the NGF gene has been tested in rat bladders ^52^. NGF levels increased in the bladder whereas bladder capacity and PVR volumes decreased, suggesting improved voiding. In the future, targeted gene therapy combined with growth factors may be beneficial in the treatment of UAB.

Stem cells also represent an exciting possible management strategy for UAB. When injected into female mice, muscle-derived stem cells have been demonstrated to differentiate into myotubes and myofibres with neuromuscular junctions in the smooth muscle layer of the bladder ^53^. These stem cells also improved the contractility of bladder strips after cryoinjury ^53^. More recently, Levanovich *et al*. ^54^ performed a pilot study of autologous muscle-derived stem cells on a single patient with UAB and unable to void. The treatment was safe, and, after 3 months, although the patient still had to self-catheterise, he was able to void small volumes with a reduction in cystometric capacity (844 to 663 ml). Stem cell therapy for UAB is undergoing a phase 2 clinical trial.

Given the poor status of current therapeutic options in established UAB, it appears that the prevention of UAB may play a role in today’s management. In patients with established risk factors for bladder dysfunction, such as diabetes mellitus or neurological disease, strict disease control may help delay the onset of UAB and DU. However, further understanding of the pathophysiology of UAB, particularly in idiopathic cases, is required before true preventative strategies may be developed.

## Future work and unanswered questions

Although there has been a recent proliferation of research into UAB, there remain a number of unanswered questions that can guide future efforts. The CURE-UAB 2 ^9^ and the ICI-RS Think Tank ^8^ identified a number of future research directions.

Clinically, a formal definition must be agreed for UAB in order to appropriately identify patients and aid future research studies. In particular, the relationship of UAB symptoms to urodynamically defined dysfunction must be established, and definitions and values for terminology must include ‘prolonged’ voiding and ‘incomplete’ bladder emptying ^8, 9^. This definition may evolve as our understanding of the natural history and disease progression of UAB increases.

The majority of current studies focus on UAB in older men; urodynamic assessments must be validated in additional patient groups, including women and other age groups ^9^. However, pressure-flow studies are invasive, and a number of authors have called for the development of non-invasive methods to characterise UAB and DU ^1, 12^.

Finally, there is a need for new potential UAB therapies. This may be established through basic science research and the identification of novel pharmacological agents or through the development of medical devices to suit the needs of patients with UAB ^8^.

## Conclusions

UAB is a complex but commonly encountered lower urinary tract condition that can significantly affect patient quality of life. At present, the aetiology and natural history, along with current management strategies for UAB, are inadequate. However, the recent proliferation of research into the condition has increased our understanding of the clinical symptoms and potential future therapies.

## Abbreviations

BOO, bladder outlet obstruction; CURE-UAB 2, 2nd International Congress of Underactive Bladder; CVA, cerebrovascular accident; DU, detrusor underactivity; ICI-RS, International Consultation on Incontinence Research Society; ICS, International Continence Society; LUTS, lower urinary tract symptom; NGF, nerve growth factor; PVR, post-void residual; SNM, sacral neuromodulation; UAB, underactive bladder.
